# Integrating the potential of ion mobility spectrometry-mass spectrometry in the separation and structural characterisation of lipid isomers

**DOI:** 10.3389/fmolb.2023.1112521

**Published:** 2023-03-16

**Authors:** Sandra M. Camunas-Alberca, Maria Moran-Garrido, Jorge Sáiz, Alberto Gil-de-la-Fuente, Coral Barbas, Ana Gradillas

**Affiliations:** ^1^ Centro de Metabolómica y Bioanálisis (CEMBIO), Facultad de Farmacia, Universidad San Pablo-CEU, CEU Universities, Urbanización Montepríncipe, Boadilla del Monte, Spain; ^2^ Departamento de Tecnologías de la Información, Escuela Politécnica Superior, Universidad San Pablo-CEU, CEU Universities, Urbanización Montepríncipe, Boadilla del Monte, Spain

**Keywords:** ion mobility spectrometry (IMS), mass spectrometry (MS), lipidomics, lipid isomers, structural isomers, stereoisomers, separation, identification

## Abstract

It is increasingly evident that a more detailed molecular structure analysis of isomeric lipids is critical to better understand their roles in biological processes. The occurrence of isomeric interference complicates conventional tandem mass spectrometry (MS/MS)-based determination, necessitating the development of more specialised methodologies to separate lipid isomers. The present review examines and discusses recent lipidomic studies based on ion mobility spectrometry combined with mass spectrometry (IMS-MS). Selected examples of the separation and elucidation of structural and stereoisomers of lipids are described based on their ion mobility behaviour. These include fatty acyls, glycerolipids, glycerophospholipids, sphingolipids, and sterol lipids. Recent approaches for specific applications to improve isomeric lipid structural information using direct infusion, coupling imaging, or liquid chromatographic separation workflows prior to IMS-MS are also discussed, including: 1) strategies to improve ion mobility shifts; 2) advanced tandem MS methods based on activation of lipid ions with electrons or photons, or gas-phase ion-molecule reactions; and 3) the use of chemical derivatisation techniques for lipid characterisation.

## 1 Introduction

**FIGURE 1:**
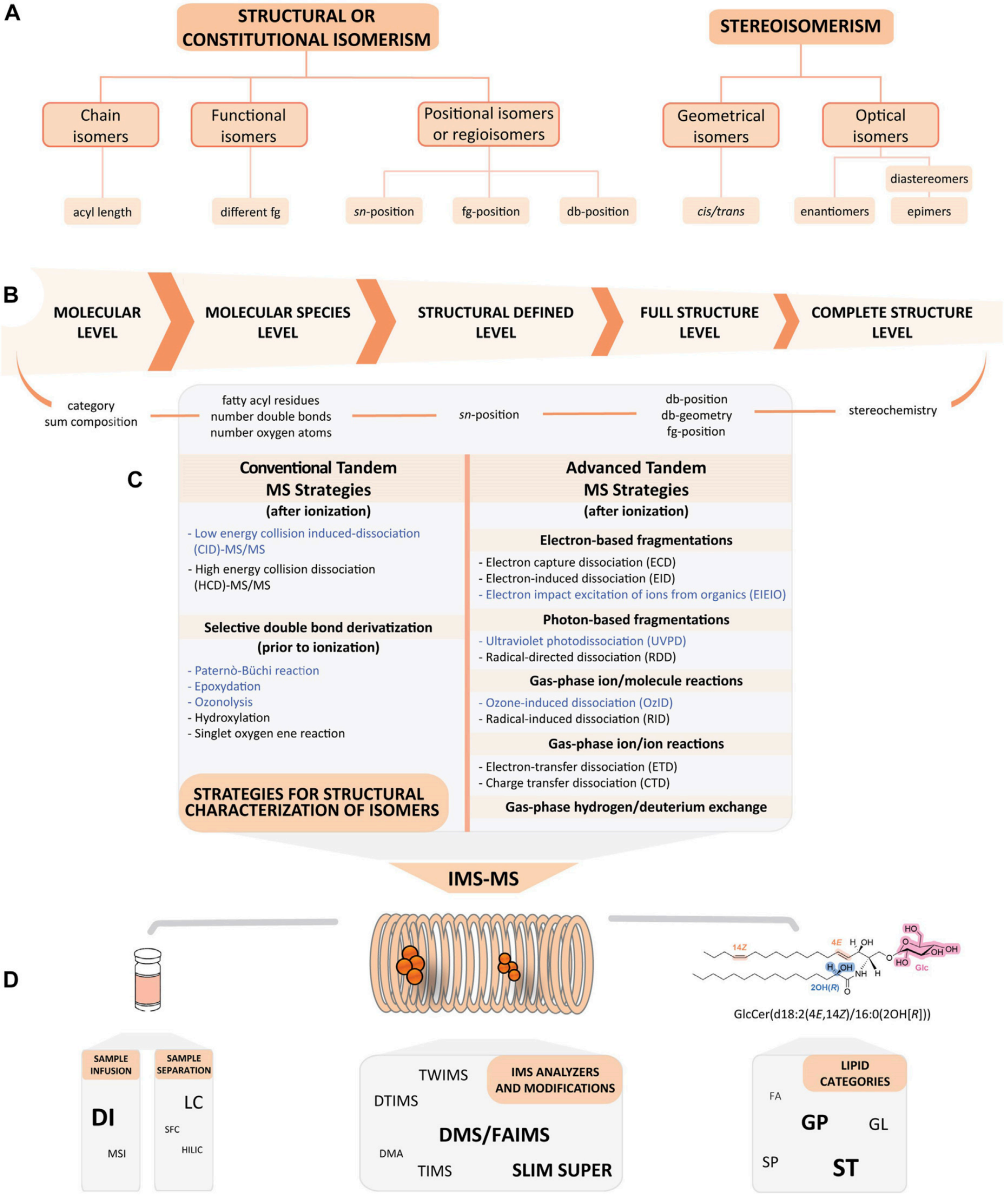
Integration of lipid isomerism resolution with structural level information and illustrative workflow of IMS-MS coupled to MS/MS techniques. **(A)** A brief outline of the five main types of isomerism that lipids can exhibit. **(B)** The structural hierarchy collected in the Lipid Map Structural Database (LMSD). Each level relates to an identification type of a specific mass spectrometry experiment (Liebisch et al., 2020). **(C)** Conventional and advanced tandem mass spectrometry methods for lipidomics, performed before or after ionisation. Highlighted in blue are the methods combined with IMS that are mainly discussed in this review. **(D)** Possibilities in the IMS-MS workflow for lipidomics. Samples can be infused directly or pre-separated by different techniques prior to ion mobility spectrometry (IMS) analysis. All lipid classes can be studied with this approach. The bold and enlarged font highlights the most exploited examples in the examples in Tables 1, 3. The molecule shown represents an example of different types of possible configurations. In orange, cis/trans isomerism; in blue, R/S isomerism; and in pink, the attachment of a carbohydrate to the lipid moiety. Abbreviations, Cer, ceramide; db, double bond; DI, direct infusion; DMA, differential mobility analysis; DMS, differential mobility spectrometry (also known as FAIMS, field asymmetric waveform ion mobility spectrometry); DTIMS, drift tube ion mobility spectrometry; FA, fatty acids; fg, functional group; GL, glycerolipids; Glc, glucose; GP, glycerophospholipids; IMS, ion mobility spectrometry; LC, liquid chromatography; MS, mass spectrometry; MSI, mass spectrometry imaging; SFC, supercritical fluid chromatography; SP, sphingolipids; SLIM, structures for lossless ion manipulation; ST, sterol lipids; SUPER, serpentine ultralong path and extended routing; TIMS, trapped ion mobility spectrometry; TWIMS, travelling wave ion mobility spectrometry.

Isomerism contributes significantly to the natural diversity of the myriad lipids that serve different metabolic functions within living organisms. The specific role of each individual lipid species is related to its chemical and physical properties, which in turn depend on the specific features of its molecular structure (Nicolau and Kokotos, 2004). The great abundance of isomeric species has made lipid characterisation particularly challenging. The several types of lipid isomers that contribute to enrich the lipidome can be broadly classified based on connectivity, into constitutional (or structural) isomers and stereoisomers (also known as spatial isomers) (Figure 1A).

Constitutional isomers share the same chemical composition but differ in the arrangement of their atoms. They are species that either have a diverse head-group composition, multiple fatty acyl/alk(en)yl chain lengths or different functional groups. Isomerism can also include alternations in the positions of fatty acids (*sn*-1, *sn*-2 and *sn*-3) on the glycerol backbone, and molecules having different functional groups and/or different carbon-carbon double-bond (db-positions) locations. These latter types are also termed positional isomers or regioisomers (Chatgilialoglu et al., 2014; Rizescu and Rizescu, 2018).

Contrastingly, stereoisomers share the same number, type of atoms, and bonds but differ in the three-dimensional orientation of their atoms in space. Stereoisomers can also show differences in their db-orientations (*cis*/*trans,* or *Z*/*E*), defined as geometrical isomers, and in the stereochemistry of the functional group (*R* vs*. S*). In this latter case, molecules can be enantiomers, if they have one chiral centre and an opposite configuration; diastereomers, with an opposite configuration at more than one chiral centre; and epimers, if they have more than one chiral centre but differ from each other in the absolute configuration at only one chiral centre (Rizescu and Rizescu, 2018) (Figure 1A).

Because of this inherent complexity, the detailed molecular structure characterisation of isomeric lipids is becoming increasingly important to comprehensively study and understand their roles in biological processes, especially so when considering the high spatial selectivity of many biochemical interactions.

The hierarchy of lipid identification based on the level of structural detail assigned to the molecule is shown in Figure 1B (Fahy et al., 2009; 2011). Tandem mass spectrometry (MS/MS) with collision-induced dissociation (CID) is the procedure of choice to probe the structural details of lipids and is thus at the core of most untargeted lipidomics studies (Wei et al., 2019; Züllig et al., 2020; Züllig and Köfeler, 2021). This approach is sufficiently powerful to determine lipid subclasses through the identification of their head-groups, their number of carbons and their degree of unsaturation of acyl/alk (en) yl chain substituents. It can also assign (although not in all cases) the chain to a specific *sn*-position (Figure 1C). Unfortunately, fragmentation-based methods lack the sensitivity needed for the unambiguous structural elucidation of lipids and are often unsuccessful in identifying isomeric variants arising from different db-positions, chain branching and cyclic structures, *cis*/*trans* geometry or chiral centres, among others.

The development of advanced tandem MS methodologies is central to resolve isomeric interferences when conventional methodologies fail. These would provide a more complete resolution and structural detail of the molecules of interest. Figure 1C summarises the advanced tandem MS methodologies available before and after ionisation using specific chemical derivatisation techniques prior to MS/MS. This includes new fragmentation modes that are aimed at improving lipid identification rates by creating more specific fragmentations, for example, electron transfer dissociation (ETD), electron-capture dissociation (ECD) or photon absorption [e.g., ultraviolet photo dissociation (UVPD)] (Zhang et al., 2022). These novel strategies have increased the level of lipid molecular information, including the characterisation of *sn*-regioisomers and db-positions, and db-stereochemistry. Several comprehensive reviews have been recently published discussing the concept, benefits, capabilities and applications of these alternatives (Hancock et al., 2017; Porta Siegel et al., 2019; Bonney and Prentice, 2021; Heiles, 2021).

As an alternative modality, ion mobility spectrometry (IMS) has provided a new paradigm in offering an important post-ionisation method for resolving gas-phase isomers before mass analysis (Mairinger et al., 2018; Paglia et al., 2021). In this context, interfacing IMS with MS (IMS-MS) has provided a superior resolution for lipids. The complementary separations in both the mobility and mass dimensions enable exceptional levels of selectivity. Accordingly, IMS-MS has emerged as a promising technique for the separation and detailed structural characterisation of lipid isomers (Kyle et al., 2016; D’Atri et al., 2018; Wu et al., 2020; Dubland, 2022) (Figure 1D).

The incorporation of the IMS-MS dimension into lipidomic workflows typically focuses on three main applications: 1) improving confidence in lipid annotation by providing mobility information of an ion as an additional descriptor (Zhang et al., 2015; Leaptrot et al., 2019); 2) reducing the complexity of mass spectra by signal filtering; and 3) enhancing the resolution of isomeric lipid species (Kliman et al., 2011; Dodds and Baker, 2019; Paglia et al., 2021). Accordingly, IMS-MS has been applied to the study of a large number of lipid classes (Damen et al., 2014; Jónasdóttir et al., 2015; Kyle et al., 2016; 2018; Cole et al., 2020; Davis et al., 2021; Duncan et al., 2021).

IMS-MS can be employed for the analysis of lipids with the extensively used direct infusion (DI) of the sample (Han and Gross, 2005), and can also be combined with mass spectrometry imaging (MSI) to visualise the spatial distribution of lipids on the sample surface (Goto-Inoue et al., 2011; Züllig and Köfeler, 2021). Developments in these approaches have been recently covered (Rivera et al., 2020).

Analytical separation platforms, of which liquid chromatography (LC) is the most used, reduce the drawbacks of DI (Jurowski et al., 2017). The most important separation technique in lipidomics is reversed-phase LC (RP-LC), but others are occasionally combined with IMS-MS, including supercritical fluid chromatography (SFC) (Xia et al., 2021) and hydrophilic interaction liquid chromatography (HILIC) (Baglai et al., 2017; Hines et al., 2017). Both are specific LC techniques that offer an alternative or complementary separation, and both have been successfully incorporated into IMS-lipidomics workflows (Li et al., 2020b).

In the next sections, we provide a selective overview of the many research studies (with emphasis on the last 5 years) where samples directly infused into IMS-MS, or used in combination with MSI, LC or SFC (including the instrumentation itself), or in combination with conventional and/or advanced tandem MS strategies, plays an integral role in separating and elucidating structural, geometrical, and optical lipid isomers. We will describe selective works using commercially available standards and mammalian samples. This includes fatty acids (FA) (e.g., oxylipins), glycerolipids (GL) [e.g., mono-, di-, and triglycerides (MG, DG, TG, respectively)], glycerophospholipids (GP) [e.g., glycerophosphocholines (PC) and lysoglycerophosphocholines (LPC), sphingolipids (SP) (e.g., sphingomyelins (SM), ceramides (Cer)], sterol lipids (ST) [e.g., steroid hormones, oxysterols, vitamin D, bile acids (BA), and gluco- and mineralocorticoids], as well as examples of the attachment of a carbohydrate to the lipid moiety [i.e., glycolipids such as gangliosides (GM)] (Figure 1D).

## 2 Separation principles of ion mobility spectrometry (IMS)

IMS is an established technique for the separation of ions based on their size and shape in gaseous phase under an electric field, which permits the separation of isomers with the same mass but different spatial configuration (Paglia et al., 2015b; D’Atri et al., 2018). IMS instruments operate with an electric field in a drift tube that drives ion movements. They also contain a buffer gas (which can be static or moving in a specific direction) that interacts with the molecules from the sample moving in the electric field. The collisions of the ions with the buffer gas separate the ions, allowing the determination of their different mobilities (K_0_), which are then used to calculate the collision cross section (CCS) of each ion by the Mason-Schamp equation (Dodds and Baker, 2019).

The CCS value is an ion-specific, highly reproducible and instrument-independent identifier. If the IMS is nested between LC and MS, the CCS values can be used as an additional parameter, together with retention time (t_R_) and *m/z*, to provide more confidence in the annotation. Having this parameter is a major advantage for lipid characterisation. Moreover, IMS reduces the spectral complexity, increases peak capacity and selectivity, and opens the door to new couplings with other techniques (Paglia et al., 2015a; May and McLean, 2015; Leaptrot et al., 2019; Luo et al., 2020). Although IMS-MS separates ions with different spatial configuration, the separation of isomers remains challenging. The differences in the spatial configuration may be minimal, resulting in a difference in CCS value (ΔCCS) <1%, which is within the error of the instrument. This makes their separation impractical in most commercial instruments due to the lack of resolution. Occasionally, analysis of commercial standards helps to visualize slight differences in mobility. Because of this, changes in the buffer gas, modified parameters or even instrumental manipulation might be necessary to enhance the separation of lipid isomers (Tu et al., 2019; Delafield et al., 2022). IMS analysers can be classified into the following three groups based on how the separation of ions occurs (May and McLean, 2015).

• Time-dispersive analysers, in which ions reach the detector at different times. This group includes drift tube ion mobility spectrometry (DTIMS), which is characterised by the separation of ions in a uniform electric field (May et al., 2014). Another manifestation is travelling wave ion mobility spectrometry (TWIMS), which uses a non-uniform electric field that creates waves to separate ions (Shvartsburg and Smith, 2008). Both approaches work with an inert, static gas that collides with the ions such that ions with smaller CCS values reach the detector first. These IMS analysers are mostly used for untargeted analysis, as all of the ionised molecules in a sample can be analysed in the same run. However, their resolution and their capacity to separate lipid isomers is limited (Moran-Garrido et al., 2022). To address this issue, and to circumvent hardware modifications, several software-based approaches have been developed to increase the resolving power in DTIMS. For instance, Agilent Technologies, Inc. commercialises a high-resolution demultiplexing software (HRdm) that is applied after data acquisition and increases resolving power.
• Confinement and selective release analysers, represented by trapped ion mobility spectrometry (TIMS). In this method, ions are trapped by an electric potential and are then released by decreasing the potential in a stepwise manner. The drift gas flows against the detector and the electric current, and ions with larger CCS values reach the detector first. These analysers can be used to separate isomers with high resolution, as the drop in the electric potential can be modified to fit the mobility of each specific ion. However, this modality is mostly used for targeted workflows. The more untargeted the approach is, the lower the resolution, and consequently the lower the separation of isomers (Jeanne Dit Fouque and Fernandez-Lima, 2019).
• Space-dispersive analysers, which include field asymmetric waveform ion mobility spectrometry (FAIMS), also known as differential mobility spectrometry (DMS). In this method, a changing voltage is applied between two electrodes as ions are transported by a carrier gas towards the detector. The ions are separated based on their mobility and only those that match the voltage applied (compensation voltage) reach the detector, which acts as an ion filter. This is the only case where the CCS values cannot be calculated, and so compensation voltages (CV) are used instead. These instruments are mostly used for targeted approaches, as only one ion can reach the detector at a time (Winter et al., 2019; Delafield et al., 2022), providing high resolution. Differential mobility analysers (DMA) are also included in this group, although they are less commonly used than FAIMS. DMA work with a constant electric field at atmospheric pressure, with ions transported by the buffer gas. They basically act as an ion filter (Dodds and Baker, 2019).

It is essential to have sufficient resolving power (R_*p*_) to separate and distinguish ions with very similar CCS values. R_*p*_ is the commonly accepted metric for quantifying the efficiency of ion mobility separation. It is defined from a single peak as a ratio of the location of the peak divided by its width (Dodds et al., 2017). New instruments and modifications to existing platforms are continually being developed to achieve better R_*p*_. For particular values of this parameter for some commercial instruments, we would refer the reader to a published review (Dodds et al., 2017). While a single-peak R_*p*_ facilitates evaluation of the performance of ion mobility instruments, separation efficiency between gas-phase ions is mostly described in terms of peak-to-peak resolution (R_*pp*_) (Dodds et al., 2017). There is some confusion in the use of these terms in the scientific community when describing IMS separations.

Several studies have attempted to separate different lipid isomers using different instruments — for example, to identify *sn*-regioisomers and db-positions in PC and LPC using DTIMS (Kyle et al., 2016). However, the limited gas-ion collisions produced under low pressure restrict the resolution of these instruments. Increasing the pressure to atmospheric or above increases the collisions between ions and the buffer gas, thus increasing the separation efficiency and R_*p*_. Accordingly, atmospheric pressure DTIMS (AP-DTIMS) has been developed to achieve better separation. These instruments can reach an R_*p*_ of up to 250, which has allowed researchers to distinguish *sn*-regioisomer and db-positional GL isomers (Groessl et al., 2015). The HRdm software by Agilent Technologies, Inc. can increase the R_*p*_ of instruments up to 350. This approach has been used to separate monoglyceride *sn*-regioisomers (May et al., 2020; da Silva et al., 2021) and steroid isomers (Dodds and Baker, 2021).

TWIMS has been used to study lipid isomers including co-eluting TG (Ferchaud-Roucher et al., 2017); however, the R_*p*_ and, consequently, the capacity to distinguish between isomers, is more limited than DTIMS (Dodds et al., 2017). As the resolution in TWIMS increases with the path length (as more collisions occur), instruments with extended paths based on travelling wave separations have been developed and commercialised (Delafield et al., 2022). These include structures for lossless ion manipulations (SLIM) with a serpentine and extended drift path (Wojcik et al., 2017), or SLIM serpentine ultralong path and extended routing (SUPER), in which the exit voltage is adjusted to promote multiple passes of ions through the drift path (Li et al., 2020a). Cyclic ion mobility spectrometry (cIMS) is another ultralong path configuration (circular path) TWIMS, in which several ion passes can occur (R_*p*_ of 750 at 100 passes) (Giles et al., 2019). Ultra-high resolution ion mobility instruments have proven their ability to separate lipid isomers including GP and TG *cis/trans* isomers (Wojcik et al., 2017; Li et al., 2020a; May et al., 2021), glycerophosphoinositols (PI) (Isaac et al., 2020), and ganglioside isomers (Isaac et al., 2022). These instruments provide better separation and higher resolution when the number of passes is increased. A different strategy that has been specifically developed for SLIM is the use of compression ratio ion mobility programming (CRIMP). This modality permits the accumulation and analysis of a larger number of ions with a higher sensitivity and resolution, as it reduces the peak broadening that commonly occurs with multiple passes of ions (Garimella et al., 2016). However, although there are several innovative ways to increase the resolution with TWIMS, they are all associated with compromised sensitivity and duty cycle.

TIMS has a higher R_*p*_ than regular DTIMS and TWIMS, at over 300 (Benigni et al., 2018; Fouque et al., 2019). Adjusting the voltages and lowering the scan rates increases the R_*p*_, allowing isomers with a ΔCCS of 0.2% to be resolved (Fouque et al., 2019). Additionally, the fragmentation mode of Parallel Accumulation-Serial Fragmentation (PASEF) permits the accumulation and fragmentation of more than one ion per scan, improving the sensitivity (Meier et al., 2018) and aiding in the elucidation of isomers by providing cleaner fragmentation spectra (Helmer et al., 2021). TIMS is becoming more widely used for isomer separation including *sn*-regioisomers and db-positions in DG and PC (Fouque et al., 2019) and isomeric glycerophosphoglycerols (PG) (Helmer et al., 2021).

Finally, space-dispersive instruments, namely, FAIMS or DMS, can reach very high R_*p*_ (around 7,900) (Santiago et al., 2015). However, because of the manner in which they work, the CCS cannot be calculated, so their R_*p*_ cannot be compared with that of other instruments. These instruments have been employed in the separation of isomeric lipids such as DG, TG, and PC, and are able to separate *sn*-regioisomers, db-positions and *cis/trans* isomers (Bowman et al., 2017). Furthermore, a better isomeric resolution can be achieved by adjusting the separation voltage, as exemplified for TG regioisomers, which were separated only when a very specific voltage was applied (Šala et al., 2016).

## 3 IMS-MS approaches towards the separation of geometrical lipid isomers by ion mobility shifts

### 3.1 Ion mobility shifts by complexation and adduct ion formation

Adduct ions are generated by the interaction between a precursor ion with one or more atoms or molecules when using soft ionisation sources (e.g., electrospray ionisation (ESI) (Murray et al., 2013). Protonated or deprotonated adducts (i.e., [M+H]^+^ and [M-H]^-^, respectively) of isomeric lipids don’t usually achieve great separation, but the formation of adducts with other molecules or complexes can cause the structures to acquire different spatial conformations, allowing their separation by IMS. The improved resolution is achieved by the shifted mobility in one of the isomeric forms because of conformational changes induced by the different coordination of the metal ion upon adduct formation (Zietek et al., 2018).

#### 3.1.1 Formation of cation adducts

**FIGURE 2:**
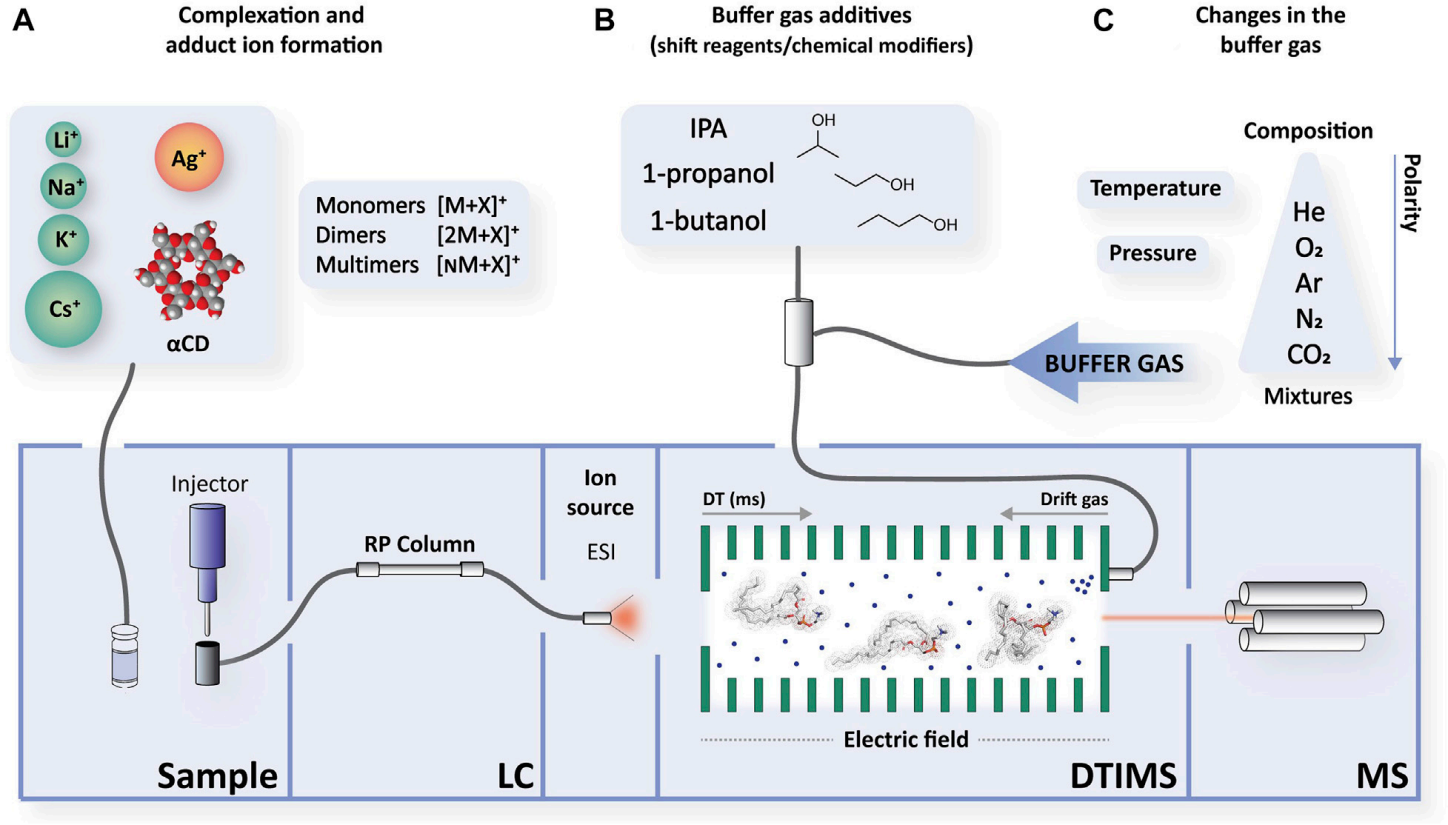
A schematic of possible changes to increase ion mobility shifts. A representative example is shown using a reversed-phase-electrospray-drift tube ion mobility spectrometry-mass spectrometry (RP-LC-ESI-DTIMS-MS) instrument. The most common modification examples employed in lipid characterisation are detailed here: **(A)** By creating different adducts upon addition of commercial salts of metals to the working solutions, or by forming complexes. Complexation and adduct formation can also occur at the same time. **(B)** By adding chemical modifiers or shift reagents (SR) that are added directly into the drift gas of the ion mobility spectrometer. **(C)** Or by modifying the composition and polarity, as well as the pressure or temperature. The drift gas enters the bottom of the mobility spectrometer with flow rates in the order of 0.5–1.5 L/min and passes through the drift tube and exits through the ionisation region. Its composition can vary depending on how it is introduced: as a single gas or as a mixture, thus modifying its polarity. Pressure, temperature, and the electric field are also configurable. Abbreviations, αCD, alpha-cyclodextrin; DT, drift time; ESI, electrospray; IMS, ion mobility spectrometry; IPA, isopropanol; LC, liquid chromatography; MS, mass spectrometry.

The most common approach to separate isomers with similar spatial conformations is the use of mono- and divalent metal cations, which form distinguishable adduct complexes with the target analytes. This can lead to successful separations of isomeric lipids and recognition of different ion conformations when using ESI in positive mode (Figure 2A). The cations (X) are added to the working solutions in the form of commercially-available salts (e.g., acetate and nitrate salts), allowing their interactions with the analytes and forcing the formation of monomers (e.g., [M+X]^+^, [M+2X]^2+^) and multimers (e.g., [2M+X]^+^ and [3M+X]^+^). The most commonly used cations for adduction are: 1) alkali metals (e.g., monovalent cations of sodium (Na^+^), potassium (K^+^), lithium (Li^+^), caesium (Cs^+^) and rubidium (Rb^+^)); 2) alkaline earth metals (e.g., divalent cations of magnesium (Mg^2+^), calcium (Ca^2+^), strontium (Sr^2+^) and barium (Ba^2+^)); and 3) transition metals (e.g., monovalent cations of silver (Ag^+^) and divalent cations of iron (Fe^2+^), cobalt (Co^2+^), nickel (Ni^2+^), copper (Cu^2+^) and zinc (Zn^2+^)).

Investigations into cation adducts have led to interesting separation results, especially with those involving alkali metals. One of the most commonly used cation adducts is sodium, which was used to successfully resolve isomeric molecules among different lipid classes. Thus, monomers of sodiated isomeric species of GL (i.e., MG and DG) have been tested in DI-DTIMS-MS (May et al., 2020) using high resolution demultiplexing (HRdm), and in LC-TIMS-MS (Fouque et al., 2019) to increase the instrument’s resolution. For instance, peaks of *sn*-positional MG isomers (i.e., 1-lineloyl glycerol (1-LG) and 2-LG) (May et al., 2020), and DG *sn*-regioisomers (e.g., DG 22:1/22:1/0:0 and DG 22:1/0:0/22:1) could be successfully differentiated (Fouque et al., 2019). While double-bond isomerism can be resolved using LC alone, IMS has a demonstrated ability of distinguishing acyl chain isomers.

A great deal of interest has been placed on PC species, on account of the abundance of isomeric species in biological samples. The position of the acyl chain on PC species plays an important role in their function, as phospholipase A2 produces lipid messengers upon cleavage of the fatty acid in the *sn*-2 position. High-resolution and multiplexing DI-DTIMS-MS has been used to separate db-positional isomers of PC with the same double bond geometries (Groessl et al., 2015). The [M+Na]^+^ adducts for PC 18:1(6*Z*)/18:1(6*Z*) and PC 18:1(9*Z*)/18:1(9*Z*) could be unequivocally detected and PC 18:1/16:0 and its acyl positional isomer could be quantified in complex extracts. Contrastingly, LC-TIMS-MS was unable to resolve the same PC molecules, as the resolution was lower for sodium adducts than for their protonated species (Fouque et al., 2019).

Steroid-hormone lipids, which comprise a wide variety of structurally related molecules, have been extensively studied in terms of their isomeric resolution because of their importance in physiological processes. Structural isomers of this group exhibit differences in the presence and position of ketone/hydroxyl groups, double bonds, and A- and D-ring functional groups. Some baseline resolved examples include dimers (i.e., [2M+Na]^+^) of corticosterone and 11-deoxycortisol by DI-TWIMS-MS (Rister et al., 2019) and, additionally, testosterone and dehydroepiandrosterone (DHEA), 17-hydroxyprogesterone and 11-deoxycorticosterone by DI-DTIMS-MS (Chouinard et al., 2017a). In the latter study, dimers showed lower resolution than their monomeric counterparts, whereas monomers showed a higher resolution for aldosterone and cortisone. Analysis of sodiated monomers in the same instrument also revealed isomer separation in human urine — for example, 7-keto-DHEA and methyl-1-testosterone (Velosa et al., 2022b).

Bile acids (BA) are steroids synthesised in the liver from cholesterol. The primary BA cholic acid, and its derivatives, exhibited measurable ion mobility differences upon investigation by DI-TWIMS (Hadavi et al., 2022). A representative isomeric pair from this study is glycodeoxycholic acid (GDCA) and glycochenodeoxycholic acid (GCDCA), whose structural differences are related to the position of the hydroxyl group on C7 and C12, respectively. The CCS values for their sodium monomers showed a difference of 8.37 Å^2^, owing to the adoption of a more planar conformation in GDCA. Conversely, multiple sodium monomers presented a bulky conformation with a poorer separation between isomers. Thus, the presence of multiple adducts does not necessarily increase their ΔCCS value (Hadavi et al., 2022).

A glycosphingolipid pair of disialoganglosides, GD1a and GD1b, which differ in the localization of sialic acid residues in their oligosaccharide head group, could only be resolved in standard mixtures with IMS-MS as doubly sodiated species [M+2Na]^2+^. DI-DTIMS-MS in combination with HRdm achieved a satisfactory peak-to-peak resolution (May et al., 2020). GD1a and GD1b have also been studied coupling an ultra-high resolution SLIM platform, which revealed distinct drift times with baseline separation even when using the minimal possible path (Wojcik et al., 2017). These promising results permitted the study of these species in more complex biological samples, including mouse brain extracts (Wormwood Moser et al., 2021).

Potassium adducts appear to have a lower resolution than sodium adducts in distinguishing the *sn*-position isomers of PC (Groessl et al., 2015). However, isomer pairs of steroid hormones such as [2M+K]^+^ adducts showed a higher resolution than sodium adducts (e.g., aldosterone and cortisone, as well as corticosterone and 11-deoxycortisol) (Rister et al., 2019).

The lithium multimeric form [2M+Li]^+^ failed to enhance resolution in DI-TWIMS-MS (Rister et al., 2019). By contrast, the [3M+Li]^+^ multimeric form of androgenic steroids (e.g., dihydrotestosterone and androsterone) could be differentiated using DI-DMS-MS (Wei et al., 2020).

Additionally, the use of lithium monomers led to significant differences in CCS values for cortisone and prednisolone in LC-DTIMS-MS with HRdm, whereas their sodium monomers were not resolved (Neal et al., 2022). Other alkali metals, such as Cs^+^ and Rb^+^, were tested in the aforementioned androgenic steroids, but the results were disappointing due to very low signal-to-noise ratios (Wei et al., 2020). Another characterisation study of glucocorticoids with the same alkali metals was also unfruitful, but in this case it was due to low resolution (Neal et al., 2022). Alternative alkaline earth metals (X^2+^) were introduced, and improvements were observed for [M+Ba+acetate]^+^ adducts (Neal et al., 2022).

Finally, it has been shown that the use of transition metals has advantages in isomer separation, as illustrated with silver, which permitted the separation of TG species as [M+Ag]^+^ adducts with exchanged fatty acyl chains in DI-DMS-MS. This method was applied to more complex biological samples of animal fats (Šala et al., 2016). Silver nitrate salt has been employed for the determination of PC 16:0/18:1 and PC 18:1/16:0 regioisomers in DI-DTIMS-MS, allowing greater differences in their K_0_ and CCS values than with Na^+^ and K^+^ metal cations (Groessl et al., 2015). First row transition metals, such as Fe^2+^, Co^2+^, Ni^2+^, Cu^2+^, and Zn^2+^ have also been tested, but the resolution did not markedly improve (Neal et al., 2022).

#### 3.1.2 Formation of anion adducts

Some examples have been reported of negatively charged molecules analysed in negative ESI mode, which helps to resolve regiosiomers; however, the lower signal obtained in most of the analyses in negative polarity limits its use. Anions can trigger conformational changes in isomers, resulting in mobility shifts between them. A good illustration of this is acetate adducts (i.e., [M+CH_3_COOH-H]^-^) of PC 18:0/20:4;OH differing in the location of the hydroxy group (i.e., C8, C9, C11, C12 or C15 position), which provided a greater drift separation than sodium adducts in LC-DTIMS-MS. While baseline separation could not be achieved, these molecules might be determined in complex samples using their CCS value. In this case, the hydroxyl group close to the head-group results in a faster mobility and a lower CCS value. This basis, either with acetate or with other ions, can be helpful when analysing PC regioisomers that differ in the position of their functional group (Hinz et al., 2019). As another example, steroids were successfully analysed with the use of chloride (Cl^−^) and fluoride (F^−^) anions in DI-TIMS, permitting the baseline resolution in prednisolone and cortisone pairs as chloride adducts (Cole et al., 2020).

#### 3.1.3 Formation of inclusion complexes

Another interesting strategy to improve separation is the formation of non-covalent binding complexes. An example is cyclodextrin acting with BA, which creates significant mobility differences among isomers. Cyclodextrin molecules are cyclic compounds composed of 6–8 glucopyranoside monomers bound in a conical fashion with a hydrophilic outer shell and a hydrophobic inner shell. Cyclodextrin is useful because the aliphatic chain of BA can be placed inside its hydrophobic cavity. Adducts of 3-amino-3-deoxy-α-cyclodextrin (αCD) (i.e., [M+αCD+H+K]^2+^) were successfully analysed in DI-TWIMS-MS SLIM SUPER together with the use of CRIMP, permitting the separation of positional isomers of taurine- and glycine-conjugated BA with sufficient resolution (Table 1, entry 23). All approaches employed in this work proved to be essential for the determination of these molecules by IMS (Chouinard et al., 2018).

**TABLE 1:** Examples of IMS-MS combined with advanced tandem mass spectrometry strategies and novel approaches in lipid analysis (i.e., derivatisation agents or complexation reagents). The distinct strategies permit isomer resolution and identification of structural and geometrical isomerism. All examples are endogenous lipids with the exception of entries 26 and 27, which are synthetic androgenic steroids. Only representative examples of isomer pair resolutions per article are listed in the table, but there might be more. Lipid standards are commercially purchased chemically pure synthetic lipid standards. Animal tissue extracts are bovine milk, porcine brain, chicken egg yolk and bovine heart. Examples were adapted to the recently published shorthand notation when possible (Liebisch et al., 2020).

Structural and geometrical isomerism						
N.	Analytical technique	Combined methods	Ion source - IMS-MS analyser	Resolved lipid isomerism	Types of samples	Ref.
Fatty acids (FA)—CLA, PUFA						
1	DI	PB-CID [^PB^M+Li]^+^	ESI-TIMS-QTOF	**db-position** CLA 18:2(9*Z*,11*E*) - CLA 18:2(10*E*,12*Z*)	Lipid standards Dietary commercial supplements	Xie and Xia (2019)
2	DI	PB-CID [^PB^M+Li]^+^	ESI-TIMS-QTOF	**db-geometry (*cis/trans*)** CLA 18:2(9*Z*,11*E*) - CLA 18:2(9*E*,11*E*)	Lipid standards Dietary commercial supplements	Xie and Xia (2019)
3	RP-LC	AMPP-CID [^AMPP^M+H]^+^	ESI-DTIMS-QTOF	**fg-position**DiHETE - HEPE - EpETE	Cell lines (Caco-2 cells) Human samples (plasma and serum)	Hellhake et al. (2020)
Glycerolipids (GL)—TG						
4	SFC	2-acpy PB-TAP CID [^PB^M+Na]^+^	ESI-TWIMS-QTOF	***sn*-position** TG 18:1(9*Z*)/16:0/18:0 - TG 16:0/18:1(9*Z*)/18:0	Lipid standards	Xia et al. (2021)
5	DI	OzID [M+Ag]^+^	ESI-DMS-QTRAP	**db-position** TG 18:1(9*E*)/18:1(9*E*)/18:1(9*E*) - TG 18:1(11*E*)/18:1(11*E*)/18:1(11*E*)	Lipid standards	Berthias et al. (2021)
Glycerophospholipids (GP)—PC, PE						
6	DI	Epoxidation [M+Li]^+^	TENG-nanoESI-TWIMS-TOF	**db-position** PC 18:1(9*Z*)/18:1(9*Z*) - PC 18:1(6*Z*)/18:1(6*Z*)	Lipid standards Animal tissue extracts	Bouza et al. (2021)
7	RP-LC	Ozonolysis [M+H]^+^	Mercury lamp + ESI-DTIMS-QTOF	**db-position** PC 18:1(9*Z*)/18:1(9*Z*) - PC 18:1(6*Z*)/18:1(6*Z*)	Lipid standards	Harris et al. (2018)
8	DI	UVPD [M+H]^+^ and [M-H]^-^	ESI-AP-DTIMS-QTRAP	**db-position** PC 18:1(6*Z*)/18:1(6*Z*) - PC 18:1(9*Z*)/18:1(9*Z*)	Lipid standards	Sanders et al. (2022)
9	DI	UVPD [M+H]^+^ and [M-H]^-^	ESI-AP-DTIMS-QTRAP	**chain isomers (acyl length)** PC 15:0/18:1(9*Z*) - PC 16:0/17:1[9-10cy3]	Lipid standards	Sanders et al. (2022)
10	DI	OzID [M+H]^+^	ESI-TWIMS-TOF	***sn*-position** PC 16:0/18:1(9*Z*) - PC 18:1(9*Z*)/16:0	Lipid standards	Vu et al. (2017)
11	DI	OzID [M+H]^+^	ESI-TWIMS-TOF	**db-geometry (*cis/trans*)** PC 18:1(9*E*)/18:1(9*E*) - PC 18:1(9*Z*)/18:1(9*Z*)	Lipid standards	Vu et al. (2017)
12	DI	OzID [M+Ag]^+^	ESI-DMS-QTRAP	**db-position and geometry (*cis/trans*)** PC 18:1(6*Z*)/18:1(6*Z*) - PC 18:1(9*E*)/18:1(9*E*)	Lipid standards	Berthias et al. (2021)
13	RP-LC	CID/OzID [M+H]^+^	ESI-TWIMS-QTOF	***sn*-position** PC 18:1(9*Z*)/18:1(9*Z*) - PC 18:1(12*Z*)/18:1(12*Z*)	Lipid standards Animal tissue extracts Olive oil	Poad et al. (2017)
14	RP-LC	CID/OzID [M+H]^+^ and [M-H]^-^	ESI-DTIMS-QTOF	**db-position** PC 18:1(9*Z*)/18:1(9*Z*) - PC 18:1(12*Z*)/18:1(12*Z*)	Lipid standards	Poad et al. (2018b)
15	RP-LC	CID/OzID [M+H]^+^ and [M-H]^-^	ESI-DTIMS-QTOF	**db-geometry (*cis/trans*)** PE 18:1(9*Z*)/18:1(9*Z*) - PE 18:1(9*E*)/18:1(9*E*)	Lipid standards	Poad et al. (2018b)
16	DI	CID/OzID [M+Ag]^+^	ESI-DMS-QTRAP	***sn*-position** PC 16:0/18:1 - PC 18:1/16:0	Lipid standards Animal tissue extracts	Maccarone et al. (2014)
17	MSI	CID/OzID [M+Na]^+^	MALDI-TWIMS-QTOF	**db-position** PC 18:1(7*Z*)/16:0 - PC 18:1(9*Z*)/16:0	Rat brain tissue	Claes et al. (2021)
Sphingolipids (SP)—SM, SPH						
18	DI	EIEIO IPA modifier [M+H]^+^	ESI-DMS + ExD cell-TOF	**chain isomers (acyl length)** SM d18:1/16:0 - SM d19:0/15:1	Animal tissue extracts	Baba et al. (2016)
19	DI	EIEIO IPA modifier [M+H]^+^	ESI-DMS + ExD cell-TOF	***sn*-position** SM d18:1/16:0 - SM d16:1/18:0	Animal tissue extracts	Baba et al. (2016)
20	DI	EIEIO IPA modifier [M+H]^+^	ESI-DMS + ExD cell-TOF	**db-position** SM d18:1/24:1;(9*E*) -SM d18:1/24:1;(6*E*)	Animal tissue extracts	Baba et al. (2016)
21	RP-LC	CID/OzID DMDS [^DMDS^M+H]^+^	ESI-DMS -QTRAP	**db-position** SPH m18:1(4*E*);3OH - SPH m18:1(14*Z*);3OH	Lipid standards Cell lines (HEK293 cells)	Steiner et al. (2016)
22	DI	OzID [M+H]^+^	ESI-DMS -QTRAP	**db-position** SPH m18:1(4*E*);3OH - SPH m18:1(6*E*);3OH	Chemically synthesised lipids	Poad et al. (2018a)
Sterol lipids (ST)—BA, SH, Chl						
23	DI	αCD [M+αCD+H+K]^2+^	ESI-TWIMS-SLIM SUPER-TOF	**fg-position** TDCA - TCDCA GDCA - GCDCA	Lipid standards	Chouinard et al. (2018)
24	RP-LC	PA-CID [^PA^M+Na]^+^	ESI-DTIMS-QTOF	**fg-position** Aldosterone - Cortisone	Lipid standards	Li et al. (2021)
25	RP-LC	PTSI [^PTSI^M-H]^-^	ESI-TWIMS-TOF	**fg-position** 22-OH-Chl - 24-OH-Chl - 27-OH-Chl	Lipid standards Cell lines (CRL-2429)	Kylli et al. (2017)
26	RP-LC	CDI [^CDI^M+H]^+^	ESI-DTIMS-QTOF	**fg-position** Methyldienolone - Boldenone	Lipid standards	Velosa et al. (2022a)
27	RP-LC	CDI+GRP [^CDI+GRP^M+H]^+^	ESI-DTIMS-QTOF	**fg-position** Methandriol - Mestanolone - Drostanolone	Lipid standards	Velosa et al. (2022a)

Abbreviations, 2-acpy, 2-acetylpyridin; αCD, alpha-cyclodextrin, AMPP, *N*-(4-amino-methyl-phenyl)-pyridinium chloride; AP-DTIMS, atmospheric pressure—drift tube ion mobility spectrometry; BA, bile acids; CDI, 1,1-Carbonyldiimidazole; Chl, cholesterol; CID, collision-induced dissociation; CLA, conjugated linoleic acids; db-geometry, double bond geometry; db-position, double bond position; DI, direct infusion; DiHETE, dihydroxy-eicosatetraenoic acids; DMDS, dimethyl disulfide; DMS, differential ion mobility spectrometry (also known as FAIMS, field asymmetric waveform ion mobility spectrometry); DTIMS, drift tube ion mobility spectrometry; EIEIO, electron impact excitation of ions from organics; EpETE, Epoxy-eicosatetraenoic acids; ESI, electrospray ionisation; ExD cell, branched radio-frequency electron-ion reaction device; fg-position, functional group position; FA, fatty acids; GCDCA, glycochenodeoxycholic acid; GDCA, glycodeoxycholic acid; GL, glycerolipids; GP, glycerophospholipids; GRP, Girard’s reagent P; HEPE, hydroxy-eicosapentaenoic acids; IPA, isopropanol; MALDI, matrix-assisted laser desorption/Ionisation; MSI, mass spectrometry imaging; OzID, ozone induced dissociation; PA, picolinic acid; PB, Paternó-Büchi reaction; PC, glycerophosphocholines; PE, glycerophosphoetanolamines; PTSI, *para*-toluene-sulfonyl isocyanate; PUFA, polyunsaturated fatty acids; QTOF, quadrupole time of flight; QTRAP, quadrupole ion trap; RP-LC, reversed-phase liquid chromatography; SFC, supercritical fluid chromatography; SH, steroid hormones; SP, sphingolipids; SLIM, structures for lossless ion manipulations; SM, sphingomyelins; *sn*-position, stereospecific numbering position; SPH, sphingosines; ST, sterol lipids; SUPER, serpentine ultra-Long path with extended routing; TAP, time-aligned parallel fragmentation; TCDCA, taurochenoxycholic acid; TDCA, taurodeoxycholic acid; TENG, triboelectric nanogenerator; TG, triglycerides; TIMS, trapped ion mobility spectrometry; TOF, time of flight; TWIMS, travelling wave ion mobility spectrometry; UVPD, ultraviolet photodissociation.

In summary, it appears that positive metal adducts provide a higher isomer resolution than negative adducts formed with negatively charged molecules or ions, regardless of their ionisation efficiency. The selection of ions to incorporate into an IMS-MS workflow for optimal separation depends strongly on the ion mobility spectrometer, the type of isomerism addressed and the analytes of interest. Currently, there is no predictive model or trend that can be reliably applied for ion selection for adduction, but the examples given in the literature may serve as a preliminary guide.

### 3.2 Ion mobility shifts upon introduction of additives (modifiers/shift reagents) in the buffer gas

Another possible IMS-MS approach to separate structurally similar lipids involves the use of additives, which can be classified as dopants and shift reagents (SR) (also known as chemical modifiers) (Fernandez-Maestre, 2018). Dopants are added in trace quantities and are introduced with the carrier gas with the purpose of reducing ionisation interferences and selectively ionising the analytes of interest. Contrastingly, SR are polar volatile molecules with free electron pairs that are injected directly into the drift region of the ion mobility spectrometer (Figure 2B). SR modify ion mobility through dynamic ion-molecule interactions as they drift through the buffer gas. In this context, the separation is achieved owing to the different structures of the ions. These differences make the ions interact differently with the SR depending on the ion and SR size, the SR-ion interaction energy (adduct stability), its concentration, intramolecular bonds, inductive effects and steric hindrance. Formation of clusters between analyte ions and SR, by an analyte dependent amount, has also been reported (Fernández-Maestre et al., 2010; Fernandez-Maestre, 2018).

Despite the large number of SR that exist (Fernandez-Maestre, 2018), only a few examples using secondary and mainly primary alcohols can be found in lipidomics analyses. For example, different polar volatile SR such as isopropanol (IPA), methanol, ethanol, 1-butanol and 1-propanol, were tested in DI-DMS-MS to separate four pairs of TG differing in their fatty acyl chain positions (Šala et al., 2016). Both 1-butanol and 1-propanol were successful, but only when combined with silver adduct formation and after carefully optimising various experimental parameters including the flow rate of the chemical modifier (Šala et al., 2016). This example shows that the sole use of additives in the drift gas is likely not sufficient to separate isomers. This finding is supported by a comparative study in which directly infused steroid hormones, using different ion mobility instruments and SR (DMA with acetonitrile and DMS with 2-propanol), showed no difference in resolution with the use of additives (Werres et al., 2019).

### 3.3 Ion mobility shifts by modifications of the buffer gas in the IMS

It is important to consider that isomeric lipid mobility shifts can be enhanced by changing the experimental variables of the buffer gas, in addition to the aforementioned approaches (Figure 2C). Separation of isomers can be achieved by buffer gas modification, although many commercial instruments do not provide this capability. These modifications include: the nature of the buffer gas, including its size, composition and polarity (Lalli et al., 2013); the gas environment, such as the temperature (Fernandez-Maestre et al., 2016) and pressure (Tabrizchi and Rouholahnejad, 2006); and the electric field conditions (Hollerbach et al., 2020).

#### 3.3.1 Modification of buffer gas composition/polarity

Nitrogen (N_2_) is the most common gas used in IMS-MS analyses (Matz et al., 2002) and the majority of the values in CCS databases have been measured with N_2_. Nevertheless, other gases of different polarities have been tested in IMS instruments (see Table 2).

**TABLE 2:** Buffer gases employed in IMS sorted by their polarity, including their polarisability constants (10-24 cm^3^) and atomic or molecular masses (amu). Data were obtained from the Computational Chemistry Comparison and Benchmark Database (CCCBDB) (Johnson, 2022). Bold fonts highlight the most commonly used gases in lipidomic analyses discussed in this review.

Buffer gas	Polarisability (10^–24^ cm^3^)	Atomic or molecular Mass (amu)
**Helium (He)**	**0.21**	**4.002602**
**Oxygen (O**_**2**_ **)**	**1.56**	**31.998060**
**Argon (Ar)**	**1.66**	**39.792000**
**Nitrogen (N**_**2**_ **)**	**1.71**	**28.012860**
Ammonia (NH_3_)	2.10	17.029950
**Carbon dioxide (CO**_**2**_ **)**	**2.51**	**44.007660**
Tetrafluoromethane (CF_4_)	2.82	88.003213
Nitrous oxide (N_2_O)	3.00	44.001189
Ethene (C_2_H_4_)	4.19	28.050560
Methyl chloride (CH_3_Cl)	4.42	50.479120
Sulphur hexafluoride (SF_6_)	4.49	146.049419

There are several examples in IMS-based lipidomics where the composition and polarity of the buffer gas have been changed. According to the data in Table 2 **,** He and Ar, although less polar than N_2_, have been implemented for some time. Helium was found to improve the separation of standards mixtures in DI-DMS-MS. An example is GL isomers with different fatty acid positions, which were resolved under He-rich gases (i.e., 70:30 He/N_2_) (Shvartsburg and Smith, 2008). However, the use of less polar gases may require other combinatory strategies such as adduct formation. For example, potassium adducts of prednisolone and cortisone in LC-DTIMS-MS in an Ar stream showed a high resolution (Neal et al., 2022).

Nevertheless, the trend seems to be the use of more polar gases. A comparative study between N_2_, He, Ar, and CO_2_ in DI-DTIMS-MS for endogenous steroid hormones concluded that more polar drift gases (i.e., CO_2_) yielded a marked improvement in mobility separation, especially for testosterone-related metabolites (Chouinard et al., 2017a). Similar results were obtained in a study comparing identical buffer gases in the same ion mobility instrumentation for cortisone and prednisolone isomers. However, in these studies other molecules showed better resolution under other gas conditions (Neal et al., 2022). Meanwhile, mixtures of gases were also tested, such as CO_2_ with breathable air (0.11% O_2_ and 79.89% N_2_). Steroids in DI-DTIMS achieved a better separation than with the use of these gases. A specific drift gas mixture worked better for some regioisomer pairs due to a better separation. For corticosterone and 21-deoxycortisol, a 55/45 CO_2_/mixture showed 85% separation) (Kaszycki et al., 2019).

As is the case with adduct formation, the separation conditions using buffer gases (i.e., used singly or as mixtures) differ depending on the isomerism tested and the pair or group of isomers examined, requiring specific and optimal configurations for each.

#### 3.3.2 Modification of buffer gas pressure

Pressure or temperature modifications are usually combined with other strategies to boost changes in mobility (e.g., combination with buffer gas additives or adduct formation). Increased pressure provides an increased number of ion-molecule collisions, thus improving the R_*p*_. An example is TG analysis using DI-DMS-MS, illustrated by the improved resolution between *sn-*regioisomers when combining silver-ion adduction, chemical modifiers and higher pressures of N_2_ (up to 41 psi) (Šala et al., 2016). Also, the same instrumentation using 35 psi of N_2_ provided a higher resolution than with lower pressures for PC regioisomers as silver adducts (Maccarone et al., 2014). Another example is the analysis of oxysterols in LC-TWIMS-MS, where up to 3.5 mbar was applied (Kylli et al., 2017). In the case of DTIMS, pressure is not easily manipulable, but commercial low- and high-pressure platforms are available. High-pressure platforms provide a higher R_*p*_ (Dodds and Baker, 2019). Up to 1,400 mbar pressure employed in high-pressure DTIMS-MS led to better separation of structural isomers of PC and gangliosides (Groessl et al., 2015; Kaszycki et al., 2019).

The temperature also influences the mobility of ions, showing an indirect relationship with resolution; that is, the lower the temperature the better the resolution achieved (Dodds and Baker, 2019). However, IMS experiments should be performed at relatively high temperatures to reduce uncertainties in the measurement of reduced mobilities (Fernandez-Maestre and Daza, 2021). Therefore, a compromise in temperature ranges must be reached to improve resolution. We were unable to find any recent lipidomic studies to highlight the temperature changes that could lead to substantial isomer resolution.

In view of the above, changes in the composition, pressure and temperature of the drift gas can be performed on most of the available IMS-MS instruments (Dodds and Baker, 2021). However, the pressure or temperature ranges vary for each instrument. Furthermore, it is likely that achieving the best separation of different lipid classes or pairs of structural lipid isomers will require specific conditions. For all these reasons, it is advisable to optimise the methods in order to obtain a broader isomer coverage in the analyses.

### 3.4 Ion mobility shifts using derivatisation methods

**FIGURE 3:**
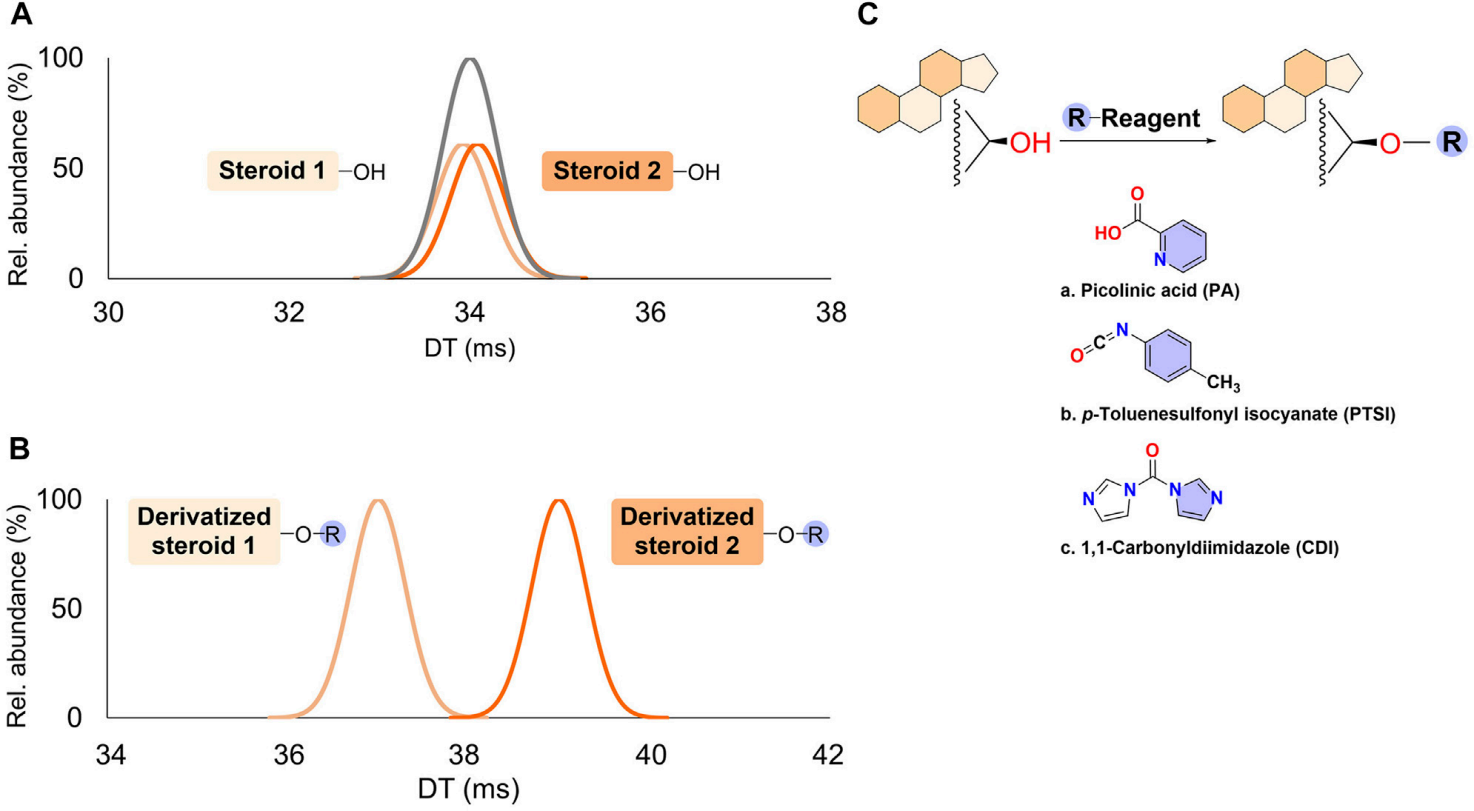
Mobility changes in steroid structural isomers with hydroxyl groups upon chemical derivatisation. **(A)** Drift times (DT) observed for two regioisomers without derivatisation. **(B)** DT observed after chemical derivatisation. **(C)** Different possibilities in steroid derivatisation described in ion mobility spectrometry-mass spectrometry (IMS-MS) workflows and discussed in the text. R refers to the derivatising reagent.

Sample derivatisation also enables the modification of the spatial conformation of isomeric molecules, leading to improved IMS resolution. This occurs through the formation of covalent bonds between derivatising reagents and oxygenated groups in the analyte (e.g., hydroxyl and carbonyl groups). The resulting conformations differ according to the reagent location, with significant differences in the CCS values in several cases. Steroids with hydroxyl groups in their four-ring core are the only lipids for which this strategy has been currently successful (Figure 3) (Velosa et al., 2022a). Derivatisation also brings the advantage of increased sensitivity in IMS-MS analysis through improved physicochemical properties of the molecules for ESI ionisation.

The use of picolinic acid (PA), which reacts with hydroxyl groups in the molecules, is a good example of a derivatisation method to increase the sensitivity and improve the separation of sterol isomers in biological samples. Using the combination of LC and DTIMS, the resolution between isomers including aldosterone and cortisone was increased with PA. In addition, ΔCCS values were widened after derivatisation, and so this parameter could be used to determine many sterols (Table 1, entry 24) (Li et al., 2021). The Li et al. study gives a promising approximation for high-throughput analyses, with the caveat that sterols that do not have hydroxyl groups will be non-reactive.

The novel derivatisation reagent *p*-toluenesulfonyl isocyanate (PTSI), which has strong nucleophilic reactivity, was able to react with hydroxyl groups and increase differences in shape, thus increasing the CCS and changing the ion-molecule interactions with gas phase molecules. In this case, oxysterols as such as hydroxy-cholesterol (OH-Chl) positional isomers 27-, 24-, and 22-OH-Chl were partially or fully separated from each other as di-PTSI derivatives in LC-TWIMS (Table 1, entry 25) (Kylli et al., 2017).

Other derivatisation strategies for steroids in IMS-MS include structurally selective reactions targeting hydroxyl (e.g., 1,1-carbonyldiimidazole (CDI)) and carbonyl (e.g., Girard’s Reagent P) functional groups. These strategies improve the ion mobility resolution and aid in structural elucidation. In a multiplexed analysis, LC-DTIMS-MS was used for studying synthetic steroids (Velosa et al., 2022a), and CDI enabled the resolution of the anabolic-androgenic steroids (AAS), boldenone and methyldienolone (Table 1, entry 26). The structural change increased the differences in CCS values and, therefore, improved the separation. Although Girard’s Reagent P (GRP) could partially separate functional group differences among steroids, its main advantage was to increase the ionisation efficiency. This was observed for 1-androstenedione and the synthetic and orally active AAS methyldienolone. CDI provokes structural changes whereas Girard’s Reagent P does not, owing to the functional group to which each bind. In the aforementioned study, a combination of both strategies and the unambiguous determination of three fg-position structural isomers based on *m/z* and CCS values was also achieved (Table 1, entry 27) (Velosa et al., 2022a). With this strategy, an “unknown” could be characterised according to its relative number of hydroxyl/carbonyl groups, illustrating the potential of this technique in the study of endogenous steroids.

As a final example to increase the sensitivity, charge-switch derivatisation with a common derivatisation reagent containing a pyridine moiety *N*-(4-amino-methyl-phenyl)-pyridinium chloride (AMPP) (Bollinger et al., 2010) permitted lipidomic analysis of oxylipins in human plasma and serum as well as in cultured cells, using a positive ionisation mode (Hellhake et al., 2020). The combination of LC-DTIMS-MS and AMPP separated functional group isomers of dihydroxy-eicosatetraenoic acids (DiHETE), hydroxy-eicosapentaenoic acids (HEPE) and epoxy-eicosatetraenoic acids (EpETE), thus enabling the characterisation of oxidised fatty acid isomers at the structural level in biological samples. This study showed more confident analysis in untargeted lipidomics, which permitted the quantification of targeted lipids (Table 1, entry 3) (Hellhake et al., 2020). Similarly, a derivatisation reaction with pyridine and thionyl was used for the simultaneous analysis of fatty alcohols, fatty aldehydes and sterols, with increased sensitivity in ESI(+) (Qi et al., 2020).

## 4 IMS-MS approaches towards the structural characterisation of geometrical lipid isomers combined with other strategies

### 4.1 Combining IMS-MS with double-bond selective derivatisation approaches (prior to ionisation)

Specific derivatisation reactions for lipid double-bonds have permitted the determination of their localisation (Zhang et al., 2022). This has been successfully implemented in IMS-MS analysis to improve the characterisation of structural isomers. Figure 1C illustrates some strategies to determine the double-bond location in lipids, and selected examples from the literature are included in Table 1. The transformations can be achieved *via* several reactions recently reviewed (Zhang et al., 2022).

The ozonolysis reactions coupled to MS have previously been used to elucidate the position of double-bonds in unsaturated lipids, as the reaction leads to diagnostic aldehyde products from the cleavage at a particular db-position. In this respect, Harris and co-workers implemented a custom-built device to perform ozonolysis reactions in the solution prior to ESI ionisation. They distinguished PC differing in db-positions; for example, PC 18:1(9*Z*)/18:1(9*Z*) and PC 18:1(6*Z*)/18:1(6*Z*) (Table 1, entry 7) (Harris et al., 2018).

Another approach of C=C selective derivatisation is the use of the classical Paternò-Büchi (PB) photochemical derivatisation reaction coupled to IMS-MS. This has been used to study db-positions in lipids by separating isomers of unsaturated lipids. The PB reaction is a cycloaddition reaction between a C=C location and a photochemically-excited carbonyl-containing compound (e.g., acetone) (Xia and Wan, 2021). Subsequent MS/MS fragmentation produces ions by cleavage at the original C=C locations with a mass shift of +58 Da, allowing the direct identification of precursor lipids (Ma et al., 2016). New halogenated acetophenones have enabled improvements in the derivatisation yield (Hynds and Hines, 2022). In particular, a PB reaction in DI-TIMS-MS followed by MS/MS spectrometry was successfully implemented to differentiate lithium adducts of conjugated linolenic acids (CLA). Lithiated adducts of PB-derivatised CLA permitted the separation of lipids differing in their db-position with different geometries, as illustrated with CLA 18:2(9*Z*,11*E*) and CLA 18:2(10*E*,12*Z*), with unique diagnostic products (Table 1, entries 1–2) (Xie and Xia, 2019).

More complex molecules, such as GL isomers, were analysed utilising charge tagging PB derivatisation, SFC and TWIMS. This combination allows fast separation of *sn-*regioisomers of DG, and separation of TG of different chain lengths and degrees of unsaturation. Time-aligned parallel (TAP) fragmentation enables multiple-stage MS/MS of the PB-derivatised lipids pinpointing the C═C location to a specific fatty acyl chain (Table 1, entry 4) (Xia et al., 2021).

An alternative application of the PB reaction may serve to localise hydroxylation sites. This would be a promising approach for the study of oxidised lipids in IMS-MS methodologies (Esch and Heiles, 2020).

The epoxidation reaction is able to pinpoint C=C locations in unsaturated lipids as a derivatisation strategy (Zhang et al., 2022). A recent study has reported the ability to structurally characterize lipids using large-area triboelectric nanogenerators (TENG) coupled with TAP fragmentation IMS-MS analysis. Gas-phase lipid epoxidation during TENG ionisation, coupled to mobility-resolved MS3 *via* TAP IMS-MS, enabled the acquisition of detailed information on the presence and position of GP C═C double bonds, the fatty acyl *sn*-chain position and composition, and the *cis*/*trans* geometrical C═C isomerism (Table 1, entry 6) (Bouza et al., 2021).

### 4.2 Combining IMS-MS with conventional tandem mass spectrometry (MS/MS) strategies (after ionisation)

The elucidation of lipids using MS/MS or MS^n^ methods relies on structural information derived from controlled fragmentation. The most common and robust conventional fragmentation mode in lipidomics to obtain tandem mass spectra is low-energy CID. This gold standard technique for characterising lipid structures functions by accelerating the precursor ions through the application of an electrical potential to increase the ion kinetic energy before collision with neutral molecules (e.g., He, Ar, N_2_). The fragment ions generated subsequently reach the detector (Sleno and Volmer, 2004).

MS/MS analyses can be classified into one of two categories: targeted or untargeted. In targeted MS/MS, the ions of interest are listed and fragmented after filtering. Conversely, in untargeted approaches, all ions are fragmented and no prior knowledge about the sample composition is needed (Moran-Garrido et al., 2022). Depending on the IMS-MS instrumentation, different approximations are adopted. While DTIMS and TWIMS are best suited for untargeted methodologies (Moran-Garrido et al., 2022); TIMS, FAIMS, DMS and DMA are best suited for targeted applications (Jeanne Dit Fouque and Fernandez-Lima, 2019; Winter et al., 2019; Delafield et al., 2022). Targeted methodologies are mainly used in the study of isomers.

The design of the instrument also defines whether the mobility information is associated with the precursor ions (when the IMS stage occurs before the precursor fragmentation) or with the product ions (when the IMS stage occurs after the precursor fragmentation). Both approaches have their advantages and disadvantages. For example, if IMS is located before the CID fragmentation (the most typical approach), the user can filter the data by the mass and the mobility of the precursor product, obtaining cleaner fragmentation spectra (Moran-Garrido et al., 2022). This combination also enables the elucidation of most *sn*-positional isomers, but also of some acyl chain isomers of DG and TG in DI-FAIMS (Bowman et al., 2017) and *sn*-regioisomers of PC in DI-FAIMS (Bowman et al., 2017) and LC-DTIMS (Odenkirk et al., 2022). PG and its regioisomer bis(monoacylglycero) phosphate (BMP) were detected using LC-TIMS instrumentation by means of PASEF (Helmer et al., 2021), in addition to two pairs of endogenous structurally related steroids using LC-DMS-MS/MS (Ray et al., 2015). Some of these studies used biological samples (Ray et al., 2015; Helmer et al., 2021; Odenkirk et al., 2022). Additionally, adduction can improve the determination of *sn*-positional isomers, such as silver-cationisation for *sn*-positional isomers of PC species by cIMS^n^ (DI-TWIMS-MS^n^) (Lillja and Lanekoff, 2022).

An interesting alternative approach is to exploit the mobility of product ions when IMS is coupled directly after the collision cell. This strategy revealed different behaviours in TWIMS for eicosanoid oxylipins (di Giovanni et al., 2018), as well as for isomeric BA (Hadavi et al., 2022). Similarly, the use of a dual-stage CID (TAP fragmentation) together with TWIMS separation of fragment ions enabled the determination of PC and LPC species (Castro-Perez et al., 2011). The measurement of the product ion mobility can be an additional and unique signature of each molecule and can be established in MS/MS experiments for more detailed information of lipids.

For a more comprehensive analysis of the use of IMS in combination with MS/MS and the different working modes that have been developed thus far, such as data dependent (DDA) or data independent acquisition (DIA) approaches, the reader can refer to a recent review published on the topic (Moran-Garrido et al., 2022).

### 4.3 Combining IMS-MS with advanced tandem mass spectrometry (MS/MS) strategies (after ionisation)

Superior methodologies of coupling ion mobility technology with advanced tandem fragmentation techniques are gradually being introduced to address isomerism forms (Figure 1C), but the use of reference standards is often required to allow unambiguous and complete structural characterisation of lipids. Table 1 summarises several combinations of IMS-MS with advanced tandem MS methods reported in the recent literature. Highlighted examples are discussed below and classified by the type of strategy performed.

#### 4.3.1 IMS-MS combined with electron-based fragmentations

Isomeric resolution by IMS-MS has been reported with alternative fragmentation methodologies, such as electron impact excitation of ions from organics (EIEIO). This has been performed in DMS analysers equipped with an ExD cell (a branched radio-frequency electron-ion reaction device) (Baba et al., 2016), and has provided structural information of lipid class, acyl length, and *sn*- and db-position of SM (Table 1, entries 18–20). The technique could also remove isobaric interferences in the combined IMS analysis of animal tissue extracts. Double-bond location was based on the presence of -2H mass shifts in the products and a characteristic “V” shape in the EIEIO fragmentation spectra (Baba et al., 2016). Accordingly, more extensive information can be obtained with the use of a single spectrum provided by this technique. The same approach was recently implemented to increase the lipid coverage when analysing GL, GP, and SP together, which allowed for the structural characterisation of over 300 regioisomer lipids in complex animal extracts (Baba et al., 2018).

#### 4.3.2 IMS-MS combined with photon-based fragmentations

Ultraviolet photodissociation (UVPD, at 193 nm) of unsaturated lipids enables a high-energy photoactivation process, resulting in the cleavage of C-C bonds adjacent to a C=C bond. This process yields diagnostic ions with a distinctive mass difference of 24 Da (Fang et al., 2020). An application of this method, coupling AP-DTIMS with a UVPD-enabled mass spectrometer and multiplexing, could unequivocally determine PC species differing in their db-locations by their UVPD spectra - for example, PC 18:1(6*Z*)/18:1(6*Z*), PC 18:1(9*Z*)/18:1(9*Z*) and PC 18:0/18:2(9*Z*,12*Z*) (Table 1, entries 8–9) (Sanders et al., 2022).

#### 4.3.3 IMS-MS combined with gas-phase ion/molecule reactions

Ozone-Induced Dissociation (OzID) involves a gas-phase reaction between ozone vapour and mass-selected unsaturated lipid ions. This yields diagnostic fragment ions (differing in 16 Da), pinpointing the db-location in the precursor ion (Xia and Wan, 2021). Several groups have explored the impact of OzID coupled to IMS-MS, as it has wide compatibility with many IMS instruments. For example, it can be coupled directly before IMS in DTIMS (i.e., the ion trap) (Poad et al., 2018b); directly to the IMS cell in TWIMS (Poad et al., 2017; Vu et al., 2017; Claes et al., 2021); or immediately after in DMS analysers (Maccarone et al., 2014; Steiner et al., 2016; Poad et al., 2018a; Berthias et al., 2021), thus modifying the mass spectrometers.

Both GL and (mostly) GP have been broadly characterised at their db-position using OzID fragmentation spectra. Some examples included lipid commercial standards of TG and PC (Table 1, entries 5 and 12). Different cations have been tested, and analyses with Ag^+^ showed the best IMS resolution and best OzID performance (Berthias et al., 2021). Moreover, isomerism was resolved for sphingosines (SPH) (Table 1, entry 22) (Poad et al., 2018a).

The backbone substitution of acyl chains and *cis/trans* isomerism have also been studied using OzID, owing to the different reaction rates between isomers. Double bonds present in *sn*-2 acyl chains and db with *trans* geometry in PC species react faster to OzID. For instance, *sn*-regioisomers PC 16:0/18:1(9*Z*) and PC 18:1(9*Z*)/16:0 differed in intensity in their OzID spectra and similar results were observed for *cis/trans* isomers PC 18:1(9*E*)/18:1(9*E*) and PC 18:1(9*Z*)/18:1(9*Z*) (Table 1, entries 10–11) (Vu et al., 2017).

Interestingly, methodologies combining OzID with CID−also known as COzID−provide more information in terms of *sn*- and db-positions and db-geometries (Table 1, entries 13–17). Some examples include PC 16:0/18:1(7*Z*) versus PC 16:0/18:1(9*Z*) (Poad et al., 2017), which were differentiated due to unequal spectra; and PE 18:1(9*E*)/18:1(9*E*) versus PE 18:1(9*Z*)/18:1(9*Z*) (Poad et al., 2018b), which showed the same spectra but different peak intensities. Another possibility is its combination with cation adduction for better IMS resolution among isomers (e.g., silver (Maccarone et al., 2014) and sodium (Claes et al., 2021) adducts). Furthermore, LC-DMS-CID/OzID was found to enable the complete characterisation of complex molecules such as an atypical 1-deoxysphingosine (SPH) after dimethyl disulfide (DMDS) derivatisation (Steiner et al., 2016) and could locate the db-position (Liao and Huang, 2022) (Table 1, entry 21).

#### 4.3.4 IMS-MS combined with gas-phase hydrogen/deuterium exchange

Hydrogen/deuterium exchange (HDX) method is based on a chemical reaction in which a covalently bonded hydrogen atom is replaced by a deuterium atom from the solvent. Its combination with DTIMS and CID fragmentation revealed that each ion exhibits a unique deuterium uptake profile (Maleki et al., 2018). In the aforementioned study, the technique did not allow the separation of isomeric GP species (e.g., PC 14:1(9Z)/14:1(9Z) and PC 14:1(9E)/14:1(9E)), but it showed a strong potential for isomer resolution upon a better understanding the behaviour of HDX (Maleki et al., 2018).

There are many other advanced tandem mass spectrometry techniques, such as those listed in Figure 1C, however, they have yet to be implemented in IMS-MS analysis. Nevertheless, many hold promise for in-depth lipid characterisation, as they have been proven successful in conventional LC-MS methodologies (Liang et al., 2007; Jones et al., 2015; Feng et al., 2019).

## 5 IMS-MS approaches towards the separation and characterisation of optical isomers by ion mobility shifts

The differentiation of optical isomers is a major challenge in the identification of lipids (Hancock et al., 2017) (Figure 1A). IMS-MS is a key approach in this respect, as the stereochemistry of a gas-phase ion will influence overall geometry and, therefore, the CCS. Although examples of resolution between enantiomers have been reported with the sole implementation of IMS to LC-MS analysis (Kaur-Atwal et al., 2011; Jónasdóttir et al., 2015; Zheng et al., 2017; Davis et al., 2021), in this section we will focus on representative examples that increase ion mobility differences (e.g., combined either with the above-mentioned adduct formation or with derivatisation).

### 5.1 Ion mobility shifts by complexation and adduct ion formation

#### 5.1.1 Formation of cation adducts

The use of adduct ions is a proven alternative for the separation of stereoisomers by altering gas-phase ion structures. New approaches in metal adduct formation with sodium in IMS-MS technologies can resolve *R* and *S* enantiomers of sphingolipids. For example, Cer 18:0;2OH[*S*] and Cer 18:0;2OH[*R*] could be discriminated using LC-DTIMS upon sodium ion binding in a standard mixture. Sodium coordinated in such a manner that the orientation of the hydroxyl group in the *R* enantiomer repelled the ceramide chains, acquiring an open conformation. Contrastingly, a closer conformation was adopted in the *S* enantiomer where the orientation was located in the opposite direction (Kyle et al., 2016).

Sodium adduct formation has been more extensively used for steroid epimers. For instance, in the case of 25-hydroxy-vitamin D3 and its epimer in carbon 3, using DTIMS (Chouinard et al., 2017b; Oranzi et al., 2018), non-conjugated muricholic acids (α-, β-, ω- and γ-MCA) and taurine conjugated muricholic acids (Tα-, Tβ-, Tω- and Tγ-MCA) using TWIMS (Hadavi et al., 2022), and androgenic steroid hormones (e.g., testosterone and epitestosterone), using TWIMS as well (Rister et al., 2019).

Due to the lack of resolution, in some cases adduction with other alkali metal has proven to be efficient, such as lithiated multimers in FAIMS for androsterone and its *trans* epimer (Wei et al., 2020). As previously stated for structural isomers, each pair of epimers requires different adducts to be resolved, and their selection must be carried out empirically. In the case of steroid hormones, extensive work has been done comparing sodium with other metal cations (potassium and lithium). The best resolution of dimers was achieved when using lithium for androsterone and epiandrosterone, potassium for α- and β-estradiol, and sodium for testosterone and epitestosterone (Rister et al., 2019).

Separation with other metal groups is both promising and challenging because of the greater number of possible adducts that can be formed and used to distinguish isomers. Alternative cation adducts have been tested for androsterone and *trans*-androsterone epimers in DTIMS, including alkaline earth metals (Mg^2+^, Ca^2+^, Sr^2+^, Rb^2+^) and first-row transition metals (Sc^3+^, Cr^3+^, Mn^2+^, Fe^2+^, Co^2+^, Ni^2+^, Cu^2+^, and Zn^2+^). A small improvement in separation was observed for alkaline earth metals, but interestingly, first-row transition metal adducts led to enhanced resolution, notably with copper and zinc adducts (Chouinard et al., 2017a).

#### 5.1.2 Formation of inclusion complexes

The previously mentioned αCD, which forms complex [M+αCD+H+K]^2+^ adducts, has also been used in epimer separation as illustrated for the BA tauroursodeoxycholic acid (TUDCA) and taurochenodeoxycholic acid (TCDCA), as well as for glycoursodeoxycholic acid (GUDCA) and glycochenodeoxycholic acid (GCDCA) in DI-TWIMS SLIM SUPER platform with the use of CRIMP software (Table 3, entry 1) (Chouinard et al., 2018).

**TABLE 3:** Examples of IMS-MS combined with advanced tandem mass spectrometry strategies and novel approaches in lipid analysis (i.e., derivatisation agents or complexation reagents). The distinct strategies permit isomer resolution and identification of optical isomerism for endogenous lipids. Lipid standards are commercially purchased chemically pure synthetic lipid standards. Only representative examples of isomer pair resolutions per article are listed in the table, but there might be more. Examples were adapted to the recently published shorthand notation when possible (Liebisch et al., 2020).

Optical isomerism						
N.	Analytical technique	Combined methods	Ion source - IMS-MS analyser	Resolved lipid isomerism	Types of samples	Ref.
Sterol lipids (ST)—BA, SH						
1	DI	αCD [M+αCD+H+K]^2+^	ESI-TWIMS-SLIM SUPER-TOF	**epimers** TUDCA - TCDCA GUDCA - GCDCA	Lipid standards	Chouinard et al. (2018)
2	RP-LC	PA-CID [^PA^M+Na]^+^	ESI-DTIMS-QTOF	**epimers** epiAN - etiocholanone	Lipid standards Mouse brain tissue	Li et al. (2021)
3	DI	PTSI [^PTSI^M-H]^-^	ESI-TWIMS-TOF	**epimers** 17α-T - 17β-T α-ES - β-ES 3α-AN - 3β-AN	Lipid standards	Ahonen et al. (2013)
4	RP-LC	PTSI [^PTSI^M-H]^-^	ESI-TWIMS-QTOF	**epimers** 7α-OH-Chl - 7β-OH-Chl	Lipid standards Human fibroblast cells	Kylli et al. (2017)
5	RP-LC	CDI [^CDI^M+Na]^+^	ESI-DTIMS-QTOF	**epimers** 17α-T - 17β-T AN - epiAN	Lipid standards	Velosa et al. (2022a)
6	RP-LC	QAO-CID [^QAO^M+H]^+^	ESI-DMS-QQQ	**epimers** 3α,5α-THP - 3β,5α-THP - 3α,5β-THP - 3β,5β-THP	Lipid standards Human samples (plasma)	Jin et al. (2013)

Abbreviations, αCD, alpha-cyclodextrin; AN, androsterone; BA, bile acids; CDI, 1,1-Carbonyldiimidazole; Chl, cholesterol; CID, collision-induced dissociation; DI, direct infusion; DMS, differential ion mobility spectrometry (also known as FAIMS, field asymmetric waveform ion mobility spectrometry); DTIMS, drift tube ion mobility spectrometry; ES, estradiol; ESI, electrospray ionisation; GCDCA, glycochenodeoxycholic acid; GUDCA, glycoursodeoxycholic acid; PA, picolinic acid; PTSI, *para*-toluene-sulfonyl isocyanate; QAO, quaternary aminooxy reagent (*O*-(3-trimethyl-ammonium-propyl) hydroxylamine bromide); QQQ, triple quadrupole; QTOF, quadrupole time of flight; RP-LC, reversed-phase liquid chromatography; SH, steroid hormones; SLIM, structures for lossless ion manipulations; ST, sterol lipids; SUPER, serpentine ultra-long path with extended routing; T, testosterone; TCDCA, taurochenoxycholic acid; THP, Tetrahydroprogesterone (also known as pregnanolone); TOF, time of flight; TUDCA, tauroursodeoxycholic acid; TWIMS, traveling wave ion mobility spectrometry.

### 5.2 Ion mobility shifts by chemical derivatisation

Derivatisation agents have been used to aid in the resolution of epimers in steroids. There are some examples of derivatised steroids whose epimer resolution appeared to be better in IMS-MS due to increased mobility differences between molecules. These agents can be combined with metal ion binding or other strategies.

Derivatisation strategies used to improve the resolution of stereoisomers are not unprecedented, as they are the same as those employed to separate structural isomers (Velosa et al., 2022a). For instance, PA permitted a better resolution between epiandrosterone and etiocholanone as [^PA^M + Na]^+^, as the ΔCCS value increased from 0.4 to 12.8 after picolinyl derivatisation and analysis in LC-DTIMS (Table 3, entry 2) (Li et al., 2021). Other examples include *p*-toluenesulfonyl isocyanate (PTSI) derivatives, whose resolution in DI-TWIMS was sufficient (peak-to-peak resolution of 0.77–1.08) for testosterone, estradiol and androsterone epimer pairs (Table 3, entry 3) (Ahonen et al., 2013). The same reagent in LC-TWIMS permitted the determination of the oxysterol epimers 7α- and 7β-hydroxy-cholesterol (OH-Chl) (Table 3, entry 4). In this case, the resolution was not sufficient, but the presence of a separated peak referring to a protomer of 7α-OH-Chl made its identification and quantification possible (Kylli et al., 2017). Finally, a recent work used new derivatisation agents to improve separation among steroid hormones. For example, CDI in combination with sodium adduction in LC-DTIMS, provided an increase of up to 15% in the ΔCCS values of the hydroxyl stereoisomer pairs epitestosterone and testosterone, as well as androsterone and epiandrosterone (Table 3, entry 5) (Velosa et al., 2022a).

There are several reagents that have solely been used for the study of epimers, such as *O*-(3-trimethyl-ammonium-propyl)-hydroxylamine bromide, a quaternary aminooxy reagent (Table 3, entry 6). This reagent reacts with the carbonyl group in C20 of neurosteroids with two chiral centres, creating a covalent bond with both possible stereochemistries *cis/trans* - as well as increasing ionisation efficiency, and therefore sensitivity, in ESI(+) analysis. The formation of molecules differing in two chiral centres - diastereomers - can be easily resolved by LC. A study using this approach achieved epimer separation (e.g., pregnanolone and allopregnanolone) in LC-DMS (Jin et al., 2013) with the conventional strategy of diastereomer formation, and implementing IMS to it.

Throughout all of the examples summarised in Table 1 and Table 3, an important limitation encountered in IMS-MS analysis is the significant necessity of commercially available reference materials, which are vital to unequivocally identify the possible lipid isomer. These can be pure chemical lipid standards or commercial mixtures that have been extracted from animal tissues, plants, or yeasts, as they often contain high amounts of specific lipids (e.g., bovine milk, heart and kidneys, porcine brain, and chicken egg yolk).

## 6 Conclusions and prospects

IMS-MS is an excellent tool for the characterisation and differentiation of lipid isomers. New strategies are continuously being developed to improve the resolving power in those cases where IMS is not sufficiently sensitive to separate the most challenging isomers, such as structural and, especially, stereoisomers of lipids. There are many diverse options available, and it is possible to combine methodologies, such as the combination of OzID with adduct formation or the use of derivatisation reagents. The latter is a promising approach for achieving full structure characterisation in lipidomic analysis and might become the future gold standard. Likewise, many of the approaches reviewed in this work could be implemented in LC-MS workflows to exploit the most advantageous features from each technique.

However, IMS-MS does has some important limitations for the characterisation of lipid isomers, which include the heterogeneity of IMS-MS as a characterisation tool for isomers, the elevated price of this technique and its associated costs, and also the lack of software for data analysis and the increased time required.

IMS-MS is continuously evolving to meet new challenges, but there remains a lack of standardised methodologies and tools for harmonised protocols for lipid isomers. For example, the variability of lipids often requires different IMS-MS methodologies for the different lipid classes, which complicates tremendously the analysis of samples. Indeed, the standardisation and simplification of methodologies is far from being realised. On the plus side, multiple approaches for the selective and sensitive characterisation of specific lipid isomers are being developed, and it seems that targeted approaches are more suitable than untargeted approaches for these characterisations. This, however, affects the laboratory throughput and uses more economic resources. Moreover, considering that some of the reviewed approaches require additional chemical reagents and/or system modifications, and bearing in mind the intrinsic high price of current IMS-MS instrumentations, the characterisation of lipids isomers with IMS-MS might be limited to its application in research and not clinical studies. Additionally, the complexity of the IMS-MS data (and the lack of bioinformatics tools) requires experienced analysts to examine and interpret the results, which also limits the use of IMS-MS. For example, very often the identification of isomer classes cannot rely solely on CCS values, as they are, typically, very similar and visual expert examination of the IMS data is needed. In this regard, the development of time-effective, versatile, and easy-to-use software solutions that provide reliable and harmonised lipid characterisation is an unmet need.

On the other hand, as described here, the separation of lipid isomers usually requires the observation of some of the least common lipid adducts, which might tremendously impact the sensitivity of the analysis. Considering the low bioavailability of some lipids and their isomers in biological samples, and the reduced sensitivity of IMS-MS compared with other MS approaches, many lipids of interest might fall below the detection limits.

The use of commercial standards has greatly facilitated the characterisation of lipid isomers by IMS-MS. They have been used to obtain experimental CCS values of isomers and are also used to determine the best conditions for the separation of isomeric forms. However, many of these isomers are not yet commercially available and their price is, typically, very high. Moreover, CCS databases, although in constant growth, still lack the relevant experimental values, especially in the context of the formation of multiple adducts, complexes and different experimental conditions such as the use of shift reagents of different buffer gases. Another reason for the heterogeneity in IMS-MS lipidomics is the fact that not all lipid classes have attracted the interest of researchers to the same extent. Although steroids are extensively studied due to their bulky conformation, there is a considerable gap in our knowledge of free or esterified oxidised lipids.

For all of the above reasons, IMS-MS is not a very common technique, and it is mostly used for basic research and method development. The use of suitable biological samples is necessary to move from this state to generate new discoveries. To date, the most promising results include the breadth of the lipid coverage through the discovery of new molecules and the creation of comprehensive CCS databases. Only a few case-control studies have so far been published in which biologically relevant isomers are characterised and could be used as potential biomarkers in disease diagnosis. In this regard, the study of oxidised lipids may well be helpful in diseases characterised by a state of oxidative stress, and IMS-MS approaches will undoubtedly be a promising tool to enrich our knowledge of these diseases.

## Glossary

αCD: 3-amino 3-deoxy alpha-cyclodextrin
AMPP: N-(4-Amino-Methyl-Phenyl)-Pyridinium Chloride
AP-DTIMS: Atmospheric Pressure DTIMS
BA: Bile Acids
BMP: Bis(monoacylglycero)phosphate
CCCBDB: Computational Chemistry Comparison and Benchmark Database
CCS: Collision Cross Section
CDI: 1,1-Carbonyldiimidazole
Cer: Ceramides
CID: Collision Induced Dissociation
cIMS: Cyclic Ion Mobility Spectrometry
CLA: Conjugated Linolenic Acids
CRIMP: Compression Ratio Ion Mobility Programming
CV: Compensation Voltage
db: Double Bond
DDA: Data Dependent Acquisition
DG: Diglycerides
DIA: Data Independent Acquisition
DHEA: Dehydroepiandrosterone
DI: Direct Infusion
DiHETE: Dihydroxy-eicosatetraenoic Acids
DMA: Differential Mobility Analysers
DMDS: Dimethyl Disulfide
DMS: Differential Mobility Spectrometry
DTIMS: Drift Tube Ion Mobility Spectrometry
EIEIO: Electron Impact Excitation of Ions from Organics
EpETE: Epoxy-eicosatetraenoic Acids
ESI: Electrospray Ionisation
ExD: Branched Radio-frequency Electron-Ion Reaction Device
FAIMS: Field Asymmetric Waveform Ion Mobility Spectrometry
FA: Fatty Acids
fg: Functional Group
GCDCA: Glycochenodeoxycholic Acid
GCDCA: Glycochenodeoxycholic Acid
GDCA: Glycodeoxycholic Acid
GL: Glycerolipids
GP: Glycerophospholipids
GRP: Girard’s Reagent P
GUDCA: Glycoursodeoxycholic Acid
HCD: High Energy Collision Dissociation
HDX: Hydrogen/Deuterium Exchange
HEPE: Hydroxy-eicosapentaenoic Acids
HILIC: Hydrophilic Interaction Liquid Chromatography
IMS: Ion Mobility Spectrometry
IMS-MS: Ion Mobility Spectrometry - Mass Spectrometry
LC: Liquid Chromatography
LPC: Lysoglycerophosphocholines
*m/z*: Mass-To-Charge Ratio
MALDI: Matrix-Assisted Laser Desorption/Ionisation
MG: Monoglycerides
MS: Mass Spectrometry
**MS/MS MS** ^**n**^ **MS** ^**2**^: Tandem Mass Spectrometry
MSI: Mass Spectrometry Imaging
OzID: Ozone-Induced Dissociation
PA: Picolinic Acid
PASEF: Parallel Accumulation Serial Fragmentation
PB: Paternò-Büchi
PC: Glycerophosphocholines
PE: Glycerophosphoethanolamines
PG: Glycerophosphoglycerols
PI: Glycerophosphoinositols
PTSI: *para*-Toluene-Sulfonyl Isocyanate
QAO: Quaternary Aminooxy Reagent (*O*-(3-trimethyl-ammonium-propyl) hydroxylamine bromide)
R*p*: Resolving Power
R*pp*: Peak-to-peak Resolution
**t**_***R***_: Retention Time
SLIM: Structures for Lossless Ion Manipulations
SP: Sphingolipids
SM: Sphingomyelins
SR: Shift Reagents
ST: Sterol Lipids
SUPER: Serpentine Ultralong Path and Extended Routing
TCDCA: Taurochenodeoxycholic Acid
TENG: Triboelectric Nanogenerator
TG: Triglycerides
TIMS: Trapped Ion Mobility Spectrometry
TUDCA: Tauroursodeoxycholic Acid
TWIM: Travelling Wave Ion Mobility Spectrometry
UVPD: Ultraviolet Photodissociation
